# Dogs and Humans Share a Common Susceptibility Gene *SRBD1* for Glaucoma Risk

**DOI:** 10.1371/journal.pone.0074372

**Published:** 2013-09-11

**Authors:** Nobuyuki Kanemaki, Kissaou T. Tchedre, Masaki Imayasu, Shinpei Kawarai, Masahiro Sakaguchi, Atsushi Yoshino, Norihiko Itoh, Akira Meguro, Nobuhisa Mizuki

**Affiliations:** 1 Veterinary Teaching Hospital, Azabu University, Sagamihara, Kanagawa, Japan; 2 Central R&D Laboratory, Menicon Co., Ltd., Kasugai, Aichi, Japan; 3 Department of Veterinary Microbiology, School of Veterinary Medicine, Azabu University, Sagamihara, Kanagawa, Japan; 4 Department of Ophthalmology, Yokohama City University School of Medicine, Yokohama, Kanagawa, Japan; Institut Jacques Monod, France

## Abstract

Glaucoma is a degenerative optic neuropathy that is associated with elevated intraocular pressure. Primary open angle glaucoma is the most common type of glaucoma in canines, and its highest incidence among dog breeds has been reported in Shiba-Inus, followed by Shih-Tzus. These breeds are known to have an abnormal iridocorneal angle and dysplastic prectinate ligament. However, the hereditary and genetic backgrounds of these dogs have not yet been clarified. In this study, we investigated the association between polymorphisms of the glaucoma candidate genes, *SRBD1*, *ELOVL5*, and *ADAMTS10*, and glaucoma in Shiba-Inus and Shih-Tzus. We analyzed 11 polymorphisms in these three genes using direct DNA sequencing. Three *SRBD1* SNPs, rs8655283, rs22018514 and rs22018513 were significantly associated with glaucoma in Shiba-Inus, while rs22018513, a synonymous SNP in exon 4, showed the strongest association (*P = *0.00039, OR = 3.03). Conditional analysis revealed that rs22018513 could account for most of the association of these SNPs with glaucoma in Shiba-Inus. In Shih-Tzus, only rs9172407 in the *SRBD1* intron 1 was significantly associated with glaucoma (*P = *0.0014, OR = 5.25). There were no significant associations between the *ELOVL5* or *ADAMTS10* polymorphisms and glaucoma in Shiba-Inus and Shih-Tzus. The results showed that *SRBD1* polymorphisms play an important role in glaucoma pathology in both Shiba-Inus and Shih-Tzus. *SRBD1* polymorphisms have also been associated with normal- and high-tension glaucomas in humans. Therefore, *SRBD1* may be a common susceptibility gene for glaucoma in humans and dogs. We anticipate that the nucleotide sequencing data from this study can be used in genetic testing to determine for the first time, the genetic status and susceptibility of glaucoma in dogs, with high precision. Moreover, canine glaucoma resulting from *SRBD1* polymorphisms could be a useful animal model to study human glaucoma.

## Introduction

Glaucoma is a degenerative optic neuropathy comprising a group of eye disorders, including visual field defects, progressive loss of retinal ganglion cells, and degeneration of optic nerve axons, and is frequently associated with elevated intraocular pressure (IOP) [1]. Glaucoma is classified into three types: primary open angle glaucoma (POAG), primary closed angle glaucoma (PCAG), and primary congenital glaucoma (PCG) [1]. POAG is the most common type of glaucoma, and is usually associated with high IOP. Japanese populations, however, have a substantially higher incidence of normal tension glaucoma (NTG), a form of glaucoma in which optic nerve damage occurs even though the IOP is not elevated [2], [3].

It is well known that glaucoma is genetically heterogeneous and many genes, such as *CYP1B1*, *MYOC*, *OPTN*, and *OPTC*, are linked to POAG and PCG in humans and/or dogs [4]–[9]. Recently, the Normal Tension Glaucoma Genetic Study Group of the Japan Glaucoma Society performed a genome-wide association study with NTG patients and controls in a Japanese population [2]. The study identified two new susceptibility genes for NTG, *SRBD1* and *ELOVL5*, with strong statistical significance. Similarly, Mabuchi et al. also reported the association of an *SRBD1* polymorphism with Japanese POAG patients, including late-onset NTG and high tension glaucoma [10].

Canine primary glaucoma has been investigated since almost 50 years ago [11], and high incidences have been reported in Beagles [12]–[14], Welsh Springer Spaniels [15], and other breeds [16], [17]. Recent study reported the Gly661Arg variant in *ADAMTS10* as the candidate disease-causing variant for POAG Beagles [12]. Kato et al. investigated the incidence of canine POAG, and reported that Shiba-Inus exhibited the highest incidence of glaucoma among 29 breeds, followed by Shih-Tzus [18]. They also reported that an abnormal iridocorneal angle and dysplastic prectinate ligament were associated with a high incidence of glaucoma in Shiba-Inus and Shih-Tzus. However, the hereditary and genetic backgrounds of glaucoma in these dogs have not yet been clarified.

In this study, to verify recent genetic findings, we investigated the association between glaucoma in Shiba-Inu and Shih-Tzu dogs and polymorphisms of glaucoma candidate genes, *SRBD1*, *ELOVL5* and *ADAMTS10*, using direct sequencing.

## Results

The average ages of glaucoma cases and controls were 8.5±2.9 and 10.0±3.0 years old, respectively, in Shiba-Inu dogs. Those in Shih-Tzu dogs were 9.2±2.0 and 10.1±2.5 years old, respectively. We genotyped 11 polymorphisms in *SRBD1*, *ELOVL5*, and *ADAMTS10* in 98 Shiba-Inu and 67 Shih-Tzu dogs using the direct DNA sequencing method (Table 1).

**Table 1:** Primer pairs for PCR of glaucoma-related genes.

Gene	Allele	5′-3′ Forward	5′-3′ Reverse
*SRBD1*	rs22019922	TGTTGGTGTTGTCAGCAAGT	TCACACTCTTTCTCTCACTCTCTC
	rs8655283	TTAGGATGAAACCATGGAAC	TTGGCGATTTATTGAACTAAC
	rs22018513, rs22018514	GCTATTGCTGATGTTGATTTG	TGCAGTGCTGGCCCTGTTGGA
	rs9172407	GTGAACCTGAAATGGCAAA	TTAACTAGCTTCCTTGCTTCC
ELOVL5	rs22226301	AGTTGTGCTGCTTACATTAGG	AGCAAGGCAAGATGTGTTC
	rs9194033	AGTGATGCTGCTATGGGATG	GCTCAGGTCATGGGATCAAG
	rs22202438	CATGCTGAACATCTGGTGGT	GCTGGTCTGGATGATTGTCA
	rs8643563	AATTGTATGGCTGGGACCAA	ACCACCAGAGGACACGGATA
	rs22194174	AATGCTTATCCTACCCAATC	TCAGGCTCTATGCTCAGTG
ADAMTS10	Gly661Arg (56097365 G>A)	CACAGAGCAAGCAGGGAGT	GGGTGGAAGTGGGAGTG

Table 2 shows the details of five single nucleotide polymorphisms (SNPs) in *SRBD1*, including their genomic locations and allele frequencies in Shiba-Inus and Shih-Tzus. In Shiba-Inus, the most statistically significant association was observed for rs22018513 (*P* = 0.00039); the G allele of rs22018513 had a 3.03-fold (95% CI = 1.62–5.65) increased risk of glaucoma, with a frequency of 78.6% in cases vs. 54.8% in controls. Significant associations were also observed for rs8655283 and rs22018514 in Shiba-Inus; the frequencies of the T allele of rs8655283 and the G allele of rs22018514 were significantly greater among glaucoma cases than among controls (rs8655283, 37.5% vs. 21.4%, *P* = 0.016, OR = 2.20, 95% CI = 1.15–4.20; rs22018514, 41.1% vs. 21.4%, *P* = 0.0037, OR = 2.56, 95% CI = 1.34–4.86). In Shih-Tzus, we observed a significant association for rs9172407 (*P* = 0.0014) and the G allele of rs9172407 had a 5.25-fold (95% CI = 1.76–15.63) increased risk of glaucoma (25.9% in cases vs. 6.3% in controls), whereas the frequency of this G allele was only moderately, but not significantly, increased in cases compared to controls in Shiba-Inus (8.9% vs. 6.0%, OR = 1.55, 95% CI = 0.51–4.71). rs22018513, which had the strongest association with glaucoma in Shiba-Inus, did not show a significant association in Shih-Tzus (*P* = 0.868, OR = 1.13, 95% CI = 0.26–4.95). Two other SNPs (rs8655283 and rs22018514), which were associated with glaucoma in Shiba-Inus, as well as rs22019922, also did not achieve statistically significant associations with glaucoma in Shih-Tzus. However, the odds-ratios of these variants in Shih-Tzus were suggestive of an association, with the T allele of rs8655283, the G allele of rs22018514, and the A allele of rs22019922, each having a 2.43 or 2.49-fold increased risk of glaucoma.

**Table 2:** Association analysis for five polymorphisms in the *SRBD1* gene region for Shiba-Inu and Shih-Tzu dog breeds.

SNP ID	Chr.	Position (CanFam2.0)	Allele	SNP Type	RiskAllele	Breed	N		Risk Allele Frequency (%)		P	OR(95% CI)
							Cases	Controls	Cases	Controls		
rs22019922	10	50924623	A/C	Intron	A	Shiba-Inu	56	42	8.9	7.1	0.65	1.27 (0.44–3.66)
						Shih-Tzu	27	40	96.3	91.3	0.25	2.49(0.50–12.49)
						Overall	83	82			0.40	1.59 (0.66–3.80)
rs8655283	10	50989281	C/T	Intron	T	Shiba-Inu	56	42	37.5	21.4	0.016	2.20 (1.15–4.20)
						Shih-Tzu	27	40	92.6	83.8	0.13	2.43 (0.75–7.89)
						Overall	83	82			0.0068	2.25 (1.28–3.97)
rs22018514	10	51,049,600	C/G	Non-synonymous	G	Shiba-Inu	56	42	41.1	21.4	0.0037	2.56 (1.34–4.86)
						Shih-Tzu	27	40	92.6	83.8	0.13	2.43 (0.75–7.89)
						Overall	83	82			0.0018	2.52 (1.43–4.44)
rs22018513	10	51,049,604	A/G	Synonymous	G	Shiba-Inu	56	42	78.6	54.8	0.00039	3.03 (1.62–5.65)
						Shih-Tzu	27	40	94.4	93.8	0.87	1.13 (0.26–4.95)
						Overall	83	82			0.0015	2.59 (1.46–4.61)
rs9172407	10	51062753	A/G	Intron	G	Shiba-Inu	56	42	8.9	6.0	0.44	1.55 (0.51–4.71)
						Shih-Tzu	27	40	25.9	6.3	0.0014	5.25 (1.76–15.63)
						Overall	83	82			0.0074	2.90 (1.34–6.26)

OR, odds ratio; CI, confidence interval.

Overall P values and ORs for meta-analysis were calculated using the Mantel-Haenzel method.

**Figure 1:**
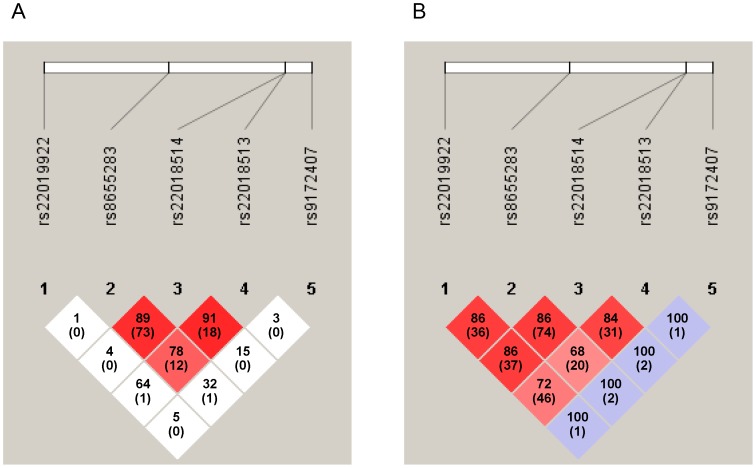
Linkage disequilibrium (LD) plot of five SNPs of the SRBD1 gene. A) LD structure in Shiba-Inus. B) LD structure in Shih-Tzus. The D’ value and r^2^ value (in parentheses) corresponding to each SNP pair are expressed as a percentage and shown within the respective square. The color scheme is based on D' and LOD score values: bright red (LOD ≥2 and D' = 1); shades of pink/red (LOD ≥2 and D' <1); blue (LOD <2 and D' = 1); white (LOD <2 and D' <1).

Figure 1 shows the strength of linkage disequilibrium (LD) for the five SNPs of *SRBD1* in Shiba-Inus and Shih-Tzus. Strong LD was observed between rs8655283, rs22018514 and rs22018513 in Shiba-Inus (D’ ≥0.78) (Figure 1A). In Shih-Tzus, strong LD was observed throughout the region from rs22019922 to rs22018513 (D’ ≥0.68) (Figure 1B). rs8655283 and rs22018514 were in almost complete LD in both Shiba-Inus and Shih-Tzus (r^2^ = 0.73 and 0.74, respectively). rs9172407 was not linked with any of the other four SNPs in either breed.

To elucidate the effect of rs8655283, rs22018514 and rs22018513 on the disease susceptibility in Shiba-Inus, we performed conditional logistic regression analysis. Conditioning by rs22018513 eliminated the significant association of rs8655283 and rs22018514, while the association of rs22018513 remained significant after conditioning by rs8655283 or rs22018514 (Table 3). These results suggest that rs22018513 could account for most of the association of these SNPs with glaucoma in Shiba-Inus.

**Table 3:** Conditional logistic regression analysis of rs8655283, rs22018514 and rs22018513 in the *SRBD1* gene for Shiba-Inus.

SNP ID				P**		
	Risk Allele	Model	P*	Covariates		
				rs8655283	rs22018514	rs22018513
rs8655283	T	Additive	0.021	–	0.92	0.15
rs22018514	G	Additive	0.0066	0.13	–	0.10
rs22018513	G	Additive	0.00025	0.0010	0.0021	–

* P values for each SNP under the recessive, additive, or dominant model that provided the best fit by logistic regression analysis. The lowest P value was selected as the best fit model. The indicated model showed the lowest P value for each SNP.

** P values adjusted for each SNP under the indicated model by conditional logistic regression analysis.

Table 4 shows the results of association analysis for the polymorphisms of *ELOVL5* and *ADAMTS10*. rs9194033 and rs22202438 of *ELOVL5* were polymorphic in Shiba-Inus and Shih-Tzus, while three *ELOVL5* polymorphisms (rs22226301, rs8643563 and rs22194174) and the *ADAMTS10* Gly661Arg variant were monomorphic. The G allele of rs9194033 and the G allele of rs22202438 had a 1.45- and a 1.39-fold increased risk of glaucoma in Shiba-Inus, respectively. Conversely, these alleles were decreased in cases compared to controls in Shih-Tzus (OR = 0.57 and 0.66, respectively). The differences in the allelic frequencies of rs9194033 or rs22202438 between cases and controls did not reach statistical significance for either breed.

**Table 4:** Association analysis for six polymorphisms in the ELOVL5 and ADAMTS10 gene regions for Shiba-Inu and Shih-Tzu dog breeds.

SNP ID	Chr.	Position (CanFam2.0)	Gene	Allele	SNP Type	Risk Allele*	Breed	N		Risk Allele Frequency (%)		P	OR (95% CI)
								Cases	Controls	Cases	Controls		
rs22226301	12	20733716	ELOVL5	C/T	3'UTR	T	Shiba-Inu	56	42	0.0	0.0	–	–
							Shih-Tzu	27	40	0.0	0.0	–	–
							Overall	83	82			–	–
rs9194033	12	20739417	ELOVL5	A/G	Intron	G	Shiba-Inu	56	42	33.9	26.2	0.25	1.45 (0.78–2.70)
							Shih-Tzu	27	40	55.6	68.8	0.12	0.57 (0.28–1.16)
							Overall	83	82			0.99	0.97 (0.61–1.54)
rs22202438	12	20743516	ELOVL5	A/G	Synonymous	G	Shiba-Inu	56	42	35.7	28.6	0.29	1.39 (0.75–2.56)
							Shih-Tzu	27	40	59.3	68.8	0.26	0.66 (0.32–1.36)
							Overall	83	82			0.98	1.02 (0.64–1.62)
rs8643563	12	20744701	ELOVL5	(–)/T	Frameshift coding	T	Shiba-Inu	56	42	0.0	0.0	–	–
							Shih-Tzu	27	40	0.0	0.0	–	–
							Overall	83	82			–	–
rs22194174	12	20749077	ELOVL5	A/C	Intron	A	Shiba-Inu	56	42	0.0	0.0	–	–
							Shih-Tzu	27	40	0.0	0.0	–	–
							Overall	83	82			–	–
Gly661Arg	20	56097365	ADAMTS10	A/G	Non-synonymous	A	Shiba-Inu	56	42	0.0	0.0	–	–
(56097365 G>A)							Shih-Tzu	27	40	0.0	0.0	–	–
							Overall	83	82			–	–

OR, odds ratio; CI, confidence interval.

Overall P values and ORs for meta-analysis were calculated using the Mantel-Haenzel method.

* Risk allele is for Shiba-Inu dogs.

## Discussion

The aim of the present study was to assess the potential associations of polymorphisms in the candidate genes *SRBD1*, *ELOVL5*, and *ADAMTS10*, with the development of canine glaucoma. To this end, we genotyped 11 polymorphisms of these genes in two breeds of dogs with cases of glaucoma or without, as controls. Here we report that the *SRBD1* polymorphisms exhibited significant association with canine glaucoma, while the *ELOVL5* and *ADAMTS10* polymorphisms that were examined in this study were not associated with canine glaucoma. In Shiba-Inus, the strongest association with glaucoma in *SRBD1* was observed at rs22018513, which is a synonymous SNP in exon 4. Two other SNPs, rs8655283 and rs22018514, were also significantly associated with glaucoma; however, these significant associations were calculated only secondarily from a strong LD with rs22018513. In Shih-Tzus, however, rs22018513 was not associated with glaucoma. Only rs9172407 in intron 1 of *SRBD1* showed a statistically significant association in Shih-Tzus, but not in Shiba-Inus. These results suggest that rs22018513 and rs9172407, or a respective neighboring polymorphism, may be a causative factor for glaucoma in Shiba-Inus and Shih-Tzus, respectively.

BLAST analysis (http://blast.ncbi.nlm.nih.gov/) reveals that the canine *SRBD1* amino acid sequence shares a high degree of similarity with its human homolog, with 90% identity. In humans, the nucleotide sequences corresponding to rs22018513 and rs9172407 are located within exon 4 and intron 1 of *SRBD1*, respectively. However, these sequences have never been reported as polymorphic in humans. While the human NTG-associated SNP, rs3213787, is located in intron 17 of *SRBD1* [2], the HapMap database shows strong LD across the entire human *SRBD1* region, suggesting that rs3213787 is strongly linked with polymorphisms in exon 4 and intron 1. Further investigation will be necessary to confirm the association of polymorphisms in the exon 4 and intron 1 regions of *SRBD1* in human glaucoma patients.

*SRBD1* was initially identified as an S1 RNA-binding domain in *Escherichia coli* [19]. Many proteins contain S1 RNA-binding domains, such as the bacterial exonuclease polynucleotide phosphorylase, eukaryotic translation initiation factor 2 alpha, and even a human DNA-binding protein [20], [21]. The function of *SRBD1* in humans and dogs remains unknown. Canine *SRBD1* is located on the reverse strand of chromosome 10, and is approximately 186 kb, including approximately 3 kb of the exon region (http://asia.ensembl.org/Canis_familiaris/Transcript/Exons?db=coreg=ENSCAFG00000002554r=10:47923593-47924593t=ENSCAFT00000049409). *SRBD1* transcript is reportedly expressed in the retinal ganglion cell and neuroblast layers in neonatal mouse tissue [2], but its localization in dogs remains unknown.

The present study found that a synonymous SNP (rs22018513) and intronic SNP (rs9172407) in *SRBD1* were associated with glaucoma in Shiba-Inus and Shih-Tzus, respectively. Synonymous and intronic polymorphisms can significantly affect gene expression by various mechanisms and lead to the development of disease [22]–[24]. We did not compare *SRBD1* mRNA levels in normal and affected dogs; however, a similar experiment has been conducted in humans [2]. Results from that study showed a significant correlation between increased *SRBD1* expression and the NTG-associated risk allele of intronic SNP. These results suggest that rs22018513 and rs9172407 in canine *SRBD1* could cause enhanced *SRBD1* expression. Reports show that *SRBD1* is indirectly involved in cell growth, general protein synthesis, induction of apoptosis, and maintaining homeostasis [2]. Therefore, we hypothesize that enhanced expression, leading to increased activity of SRBD1, which could induce apoptosis, could result in retinal ganglion cell death during the development of glaucoma.

ELOVL5 is a fatty acid condensing enzyme involved in the biosynthesis of long-chain polyunsaturated fatty acids [25], and is one of the candidate genes for retinitis pigmentosa [26]. The Normal Tension Glaucoma Genetics Study Group of the Japan Glaucoma Society reported *ELOVL5* as a new susceptibility gene for human NTG [2]. However, the present study did not show any significant association of *ELOVL5* polymorphisms with canine glaucoma. The difference between our results and those reported by the study group may be due to the different forms of glaucoma that were studied (human NTG without IOP elevation, and canine glaucoma with IOP elevation, respectively). In contrast, *SRBD1* polymorphisms were associated with canine glaucoma and human glaucoma independent of IOP, suggesting that *SRBD1* polymorphisms may affect a common disease condition in canine and human glaucoma. Therefore, detection of any common phenotypes in these glaucoma studies [2], [10] is important because it will help to clarify how *SRBD1* affects the development of glaucoma. Moreover, canine glaucoma resulting from *SRBD1* polymorphisms could be used as an excellent genetic animal model for human glaucoma and contribute significantly to the development of novel diagnostic and therapeutic options for glaucoma.

Kato, et al., reported that the Beagle breed has the fourth-highest incidence of canine glaucoma, after Shiba-Inu, Shih-Tzu, and American Cocker Spaniel breeds [18]. Kuchtey et al. recently reported that the Gly661Arg variant (56097365 G>A) of *ADAMTS10* in Beagles with POAG is a candidate, predictive gene allele for canine POAG [12]. We did not observe an association between this 56097365 G>A variant and glaucoma in Shiba-Inus or Shih-Tzus, possibly because of breed-specific allelic differences between Beagles and Shiba-Inus or Shih-Tzus. More recently, Kuchtey, et al., reported that the Gly661Arg variant was not found in any of the other dog breeds analyzed, (Shiba-Inu, Shih-Tzu, American Cocker Spaniels, Chihuahua, Australian Cattle Dog, Jack Russell Terrier, Jindo, Siberian Husky, and Yorkshire Terrier), suggesting that this allele is Beagle-specific, and that other genes may be associated with glaucoma in other breeds [27]. However, *ADAMTS10* may be still a candidate gene for glaucoma in dogs, including Shiba-Inu and Shih-Tzu, because other *ADAMTS10* variants have yet to be investigated for their association with canine glaucoma. The Ensembl database (http://asia.ensembl.org/index.html) shows 16 genetic polymorphisms in the canine *ADAMTS10* gene region. Since the dog genome information is still incomplete, it is predicted that more even polymorphisms exist in the canine *ADAMTS10* gene region. Therefore, it is necessary to perform a comprehensive genetic analysis of the region and clarify whether *ADAMTS10* is a candidate gene for glaucoma not only in Beagles, but also in other breeds.

There are no genetic tests currently available to assist in glaucoma diagnosis, identification of people at risk, initiation of treatment, and timing of surgical intervention. We performed the present SNP analysis of candidate genes in Shiba-Inu and Shih-Tzu dog breeds for the possibility to help develop diagnostic, genetic analyses for glaucoma risk factors. However, the mechanism by which these genes contribute to the development of glaucoma remains to be determined. Future studies are expected to examine the roles of *SRBD1* in humans and dogs, in an effort to determine whether genetic testing might not only help predict whether someone will develop glaucoma, but may also, perhaps, be a valuable prognostic factor for the clinical course of the disease, and/or predictive factor for its treatment. Despite new and improving diagnostic and therapeutic options for glaucoma, blindness resulting from glaucoma remains a major public health problem. These future experiments will help to optimize glaucoma treatment.

## Materials and Methods

### Ethics Statement

This study was performed as part of research approved by the Ethical Committee of Azabu University (Permit Number: 110408-2). Informed written consent was obtained from each dog owner. All procedures in this study were conducted in accordance with the Guide for the Care and Use of Laboratory Animals of Azabu University.

### Diagnosis of Glaucoma

98 Japanese Shiba-Inu dogs and 67 Shih-Tzu dogs were recruited from the Veterinary Teaching Hospital at Azabu University. All dogs received complete ophthalmologic examinations using a hand-held slit-lamp biomicroscope (SL-14; Kowa, Tokyo, Japan), indirect ophthalmoscopy, and tonometry. After the application of topical anesthesia (oxybuprocaine hydrochloride, Santen, Osaka, Japan), IOP was measured by tonometry using the Tono-Pen XL (Mentor O&O Inc., Norwell, MA). 42 Shiba-Inus and 40 Shih-Tzus were diagnosed as normal (<25 mmHg IOP). 56 Shiba-Inus and 27 Shih-Tzus had elevated IOP (>25 mmHg) in at least one eye, and were diagnosed with glaucoma. Since glaucoma is a late-onset disorder, we did not recruit dogs younger than four years in the control group, in an attempt to exclude potential glaucomatous dogs.

### DNA Preparation

Genomic DNA from glaucomatous and normal dogs was collected from peripheral blood and purified using a DNA whole blood spin kit (Fuji Film, Tokyo, Japan). The purity and concentration of DNA were examined using GeneQuant Pro (GE Healthcare, Cambridge, UK).

### Determination of DNA Sequences

In the *SRBD1* and *ELOVL5* gene regions, a total of ten polymorphisms were selected to cover the entire gene regions (rs22019922, rs8655283, rs22018514, rs22018513 and rs9172407 in *SRBD1*; rs22226301, rs9194033, rs22202438, rs8643563 and rs22194174 in *ELOVL5*) (Table 2,4). In the *ADAMTS10* gene region, we selected the Gly661Arg variant (56097365 G>A) for analysis (Table 4). PCR primer pairs listed in Table 1 were used to amplify regions containing the SNPs mentioned above. The PCR products were electrophoretically separated on a 1% agarose gel, and the PCR product bands were cut and frozen at −80°C in Tris-EDTA buffer. The frozen samples were thawed, homogenized, and centrifuged at 15,000 *g* for 5 minutes. The supernatants were subjected to cycle sequencing using the Big Dye terminator sequencing kit (Life Technologies, Foster City, CA). The sequence cycling reaction products were purified using the Qiagen Dye Ex 2.0 Spin Kit (QIAGEN, Hilden, Germany) according to the manufacturer’s instructions. After drying, the DNA samples were mixed with high Dye Mix (Life Technologies) and were directly sequenced using an ABI PRISM 310 genetic analyzer (Life Technologies). The sequence data were analyzed using Sequence Scanner v. 1.0 (Life Technologies) and GENETYX-WIN v. 4.0 (Genetyx, Tokyo, Japan). We did not find any novel DNA sequences deposited in GenBank in this study.

### Statistical Analysis

Hardy-Weinberg equilibrium was tested for each SNP among glaucomatous and normal dogs. Differences in allele frequency between glaucomatous and normal dogs were assessed using the χ^2^ test, and *P*<0.05 was considered statistically significant. Overall, *P* values and odds ratios (ORs) for meta-analysis were calculated by the Mantel-Haenszel method. The Haploview 4.1 program was used to compute pairwise LD statistics [28]. Conditional logistic regression analysis was performed to assess the effect of each SNP on the disease susceptibility using PLINK (http://pngu.mgh.harvard.edu/purcell/plink/) [29].
